# Evaluation of a Digital Media Campaign to Promote Knowledge and Awareness of the GPFirst Program for Nonurgent Conditions: Repeated Survey Study

**DOI:** 10.2196/66062

**Published:** 2025-04-14

**Authors:** Rebecca Ong Hui Shan, Hong Choon Oh, Priscilla Goh Sook Kheng, Lyndia Lee Sze Hui, Mas Riza Bte Mohd Razali, Edris Atikah Ahmad, Jagadesan Raghuram, Choon How How, Steven Lim Hoon Chin

**Affiliations:** 1 Health Services Research Changi General Hospital SingHealth Singapore Singapore; 2 Centre for Population Health Research and Implementation SingHealth Singapore Singapore; 3 Primary Care Integration Changi General Hospital SingHealth Singapore Singapore; 4 Chairman Medical Board's Office Changi General Hospital SingHealth Singapore Singapore; 5 Family Medicine Academic Clinical Programme Duke-NUS Singapore Singapore; 6 Department of Emergency Medicine Changi General Hospital SingHealth Singapore Singapore

**Keywords:** digital media campaign, public awareness campaign, primary care partnership, social media, nonurgent emergency department visits, Andersen model

## Abstract

**Background:**

GPFirst is a primary care partnership program designed to encourage patients with nonurgent conditions to seek care at participating general practitioner clinics instead of visiting the emergency department. In 2019, a digital media campaign (DMC) was launched to raise awareness and knowledge about GPFirst among residents in eastern Singapore.

**Objective:**

This study aims to assess the DMC’s impact on awareness and knowledge of GPFirst across different age groups, and the acceptability and satisfaction of GPFirst.

**Methods:**

The DMC, comprising Facebook posts and a website designed using the Andersen behavioral model, was evaluated through 2 repeated cross-sectional surveys. The first cross-sectional survey (CS1) was conducted with eastern Singapore residents aged 21 years and older, 2 1 year before the campaign’s launch, and the second survey (CS2) 4 months after. Satisfaction was measured on a 5-point Likert scale (very poor to excellent) about GPFirst experiences. Acceptability was assessed with 3 yes or no questions on decisions to visit or recommend GPFirst clinics. Analyses used tests of proportions, adjusted multiregression models, and age-stratified secondary analyses.

**Results:**

The Facebook posts generated 38,404 engagements within 5 months, with “#ThankYourGP” posts being the most viewed (n=24,602) and engaged (n=2618). Overall, 1191 and 1161 participants completed CS1 and CS2 respectively. Compared to CS1, CS2 participants were more aware (odds ratio [OR] 2.64, 95% CI 2.11-3.31; *P*<.001) and knowledgeable of GPFirst (OR 4.20, 95% CI 2.62-6.73; *P*<.001). Awareness was higher among married individuals (OR 1.31, 95% CI 1.04-1.66; *P*=.03), those without a regular primary care physician (OR 1.79, 95% CI 1.44-2.22; *P*<.001), and with higher education levels. Similarly, knowledge was greater among individuals with secondary (OR 2.88, 95% CI 1.35-6.17; *P*=.006) and preuniversity education (OR 2.56, 95% CI 1.14-5.70; *P*=.02), and those without a regular primary care physician (OR 1.54, 95% CI 1.02-2.34; *P*=.04). For acceptability, among participants who visited a GPFirst clinic, 98.2% (163/166) reported they would continue to visit a GPFirst clinic before the emergency department in the future, 95.2% (158/166) would recommend the clinic, 60.2% (100/166) cited the clinic’s participation in GPFirst as a factor in their provider’s choice and 87.3% (145/166) were satisfied with GPFirst. Among those unaware of GPFirst, 88.3% (1680/1903) would consider visiting a GPFirst clinic before the emergency department in the future.

**Conclusions:**

The DMC improved awareness and knowledge of GPFirst, with high satisfaction and acceptability among participants. Age-dependent strategies may improve GPFirst participation. The “#ThankYourGP” campaign demonstrated the potential of user-generated content to boost social media engagement, a strategy that international health systems could adopt.

## Introduction

Overcrowding in emergency departments (EDs) is a global concern, exacerbated by nonurgent patient visits [1-3], which could be managed in primary care settings. In the United States, studies consistently report nonurgent ED visit rates exceeding 30% [3], leading to adverse outcomes such as medication errors and increased mortality [4,5]. Redirecting these patients to primary care providers could potentially mitigate health care costs [6], improve patient safety and optimize ED resource allocation for urgent cases. Singapore faces a parallel challenge, with nonurgent visits, defined as mild-to-moderate conditions that could be managed in primary care, contributing to over 50% of cases in public hospitals as of 2013 [7]. In response, the GPFirst primary care partnership program was launched in 2014 at Changi General Hospital’s (CGH) ED department [8]. GPFirst is an educational program that integrates financial incentives to encourage patients with nonurgent conditions to seek initial care at participating GPFirst clinics. The program educates the public on recognizing these nonurgent conditions. Additionally, GPFirst fosters collaboration with general practitioners to enhance the management of nonurgent conditions in primary care. To reinforce these educational efforts, the program offers a financial incentive in the form of an SG $50 (US $36.50) discount on ED fees for patients who first seek care at participating GPFirst clinics and subsequently require a referral to CGH’s ED. Research indicated that this amount adequately covers most primary care expenses [9].

Recognizing the need to enhance awareness and understanding of GPFirst among the 1 million residents within its catchment area [10], CGH initiated a digital media campaign (DMC) in 2019. The campaign aimed at promoting visits to participating GPFirst clinics over ED visits for nonurgent health issues. This was done through the dissemination of educational materials via social media and a website, which provided information on nonurgent conditions manageable by general practitioners, and the location of participating GPFirst clinics. The GPFirst team selected digital media (DM) for the campaign due to its proven ability to reach diverse demographic groups and promote health-related behavioral changes through integrated health promotion content, as highlighted by existing literature [11-19].

In Singapore, individuals aged 39 years and younger account for over 50% of nonurgent visits, with the 40-59 years age group contributing 25%, and those aged 60 years and older constituting 15.2% [20]. A 2019 national survey highlighted that 88% of adults aged 25 years and older use the internet for the prevalent activity of social networking [21]. Particularly, Facebook is popular among individuals aged 25-44 years, with approximately 57% actively using the platform in 2019 [22]. This offered an opportunity to leverage digital platforms like Facebook to disseminate health educational material and catalyze health behavior changes [23,24].

While numerous studies have explored the use of DM in promoting healthier lifestyle practices [15,25], gaps remain in understanding its impact in promoting specific health program awareness and knowledge and how this impact may vary across diverse age groups. Addressing these gaps is also crucial for advancing health equity [26,27], as awareness and understanding of health programs can vary significantly among different demographic segments, which could lead to unequal access to its benefits. Younger individuals or those with higher education levels might be more adept at finding and understanding health care information, whereas older adults or those with lower health literacy may be less aware of the program’s benefits [28].

This study primarily aims to assess the impact of a new DMC on the awareness and knowledge of GPFirst among residents who reside in eastern Singapore. The study also has a secondary aim of assessing the overall acceptability and satisfaction of GPFirst within this community since its introduction in 2014.

## Methods

### Overview

The roll-out of DMC entailed two key phases, namely (1) campaign development and (2) impact assessment. Details of these 2 phases are described in the following sections.

### DMC Development

To capitalize on the popularity of Facebook and the internet in the general local population, DMC leveraged information-seeking models [29] that delineate 2 primary mechanisms for acquiring digital health information: purposive information-seeking and information encountering. While purposive seeking involves intentional efforts to obtain specific information, information encountering occurs incidentally during routine digital activities [29]. Research shows that individuals frequently encounter health-related information incidentally more than they actively seek it out [30,31]. The study team postulated that placement of GPFirst-related information on frequently visited DM platforms increases the likelihood that target residents would encounter messages promoting GPFirst during their routine digital activities. This approach could potentially enable the DMC to reach a broad audience, including those who may not actively seek out health information but could benefit from the program’s messages.

The development of the DMC entailed a collaborative effort among a multidisciplinary team from CGH, including ED and community care practitioners, researchers, data managers, corporate communications personnel, GPFirst program executives, and a communication agency (GOLIN/HARRIS International Pte Ltd). This agency was commissioned to create the educational materials featured in this study, with actors and models specifically hired for their production. All images, videos, and content are owned and protected under CGH’s intellectual property rights. Furthermore, the Primary Care Advisory Council, comprising health care administrators and clinicians dedicated to advancing primary health care delivery models and initiatives in eastern Singapore, provided advisory support on the campaign’s content and branding.

The objective of this stage was to generate content aimed at fostering community understanding and recognition of the legitimacy of ED overcrowding, while also delivering educational material essential for behavioral change. This approach is rooted in established behavior change models such as the Andersen behavior model [32-34]. According to the Andersen model, the use of health services is influenced by 3 key components: predisposing factors (personal characteristics and beliefs affecting service use likelihood), enabling factors (resources that facilitate or impede health care access), and need factors (individual perception of health needs driving care-seeking decisions) [32-34]. In the context of individuals who have nonurgent conditions, we postulated that they are less likely to visit the ED if (1) they perceive their general practitioners as capable of managing their health conditions and understand that EDs are reserved for urgent cases (predisposing factors), (2) they are educated on identifying nonurgent conditions and have access to participating GPFirst clinics (enabling factors), and (3) they are able to comprehend the perceived severity of their health condition (need factors). As such, the campaign content was developed so that it was embedded adequately with the aforementioned predisposing, enabling, and need factors. The development process involved regular weekly meetings to craft campaign messaging, educational content, and design elements. This collaborative endeavor led to the creation of 2 digital platforms aimed at promoting GPFirst, detailed in the subsequent sections.

### GPFirst Facebook Page

The GPFirst Facebook page [35] adopted a targeted strategy to engage its social media audience by sharing 2-3 posts weekly from end-July 2019 to December 2020. The campaign included informative content (Multimedia Appendix 1), video reels, education on various nonurgent conditions, festival greetings, regular quizzes, and special giveaway events like the “8 Weeks of Gifting,” where participants could win prizes by correctly answering questions posted on the GPFirst Facebook page. These activities are aimed at engaging and educating the audience, addressing the Andersen predisposing factors by shaping beliefs and attitudes toward using general practitioner services over EDs. An event titled “#ThankYourGP” encouraged individuals to share personal stories and show appreciation for their general practitioners (Multimedia Appendix 1), with a chance to win a nonmonetary prize. This initiative was intended to build trust and positive perceptions of general practitioners, further addressing predisposing factors.

**Figure 1:**
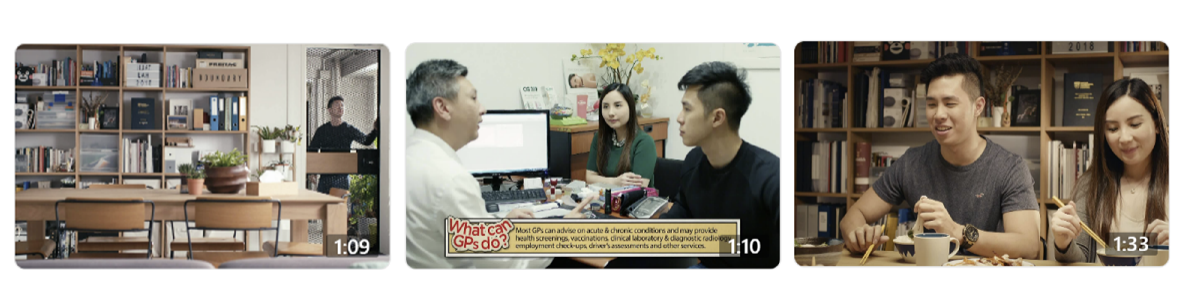
Zac and Cassy 3-part video series. The individuals in the images are paid models or actors hired by Golin Pte Ltd. The intellectual property rights for all designs and deliverables (images/videos) produced by the vendor (Golin Pte Ltd) belong to Changi General Hospital, and are used here with permission.

GPFirst leveraged the Facebook platform to share educational videos about the appropriate use of ED services. Each video was crafted to highlight the nonurgent conditions manageable by general practitioners, emphasizing the Andersen enabling factors by informing the audience about available resources and appropriate health care access points. One notable concept was a 3-part series featuring live actors (Figure 1). This series followed the story of a youth named Zac, who, having a fever, opted to visit the ED accompanied by his sister (Cassy). Zac and Cassy observed individuals in the ED with conditions that did not warrant emergency care. Through Cassy’s guidance, Zac learned that general practitioners are a viable alternative for such conditions and discovered the benefits of GPFirst.

The narrative of Zac’s story informed users of the inappropriate use of the ED for minor ailments like mild fevers and colds, addressing the Andersen need factors by helping individuals understand the severity of their health conditions and the appropriate care settings. The series concluded with Zac and Cassy returning home to share their newfound knowledge about GPFirst with their parents and neighbors, thereby spreading awareness within their community and reinforcing the Andersen enabling factors by promoting access to GPFirst services.

### GPFirst Website

GPFirst website [36] was designed to address Andersen needs and enabling factors. The website offers both an overview and a detailed explanation of GPFirst, emphasizing its purpose and benefits. The smartphone-friendly design ensured a seamless browsing experience on smartphones and tablets. The homepage introduces visitors to GPFirst Facebook videos and provides links to numerous other educational materials and resources. A dedicated section titled “Conditions your GP can treat” provides an extensive list of various medical conditions, indicating whether they necessitate a visit to the general practitioner, ED, or managed at home. This helps visitors determine the appropriate course of action based on the presented symptoms, aligning with the need factors of the Andersen model. Enabling factors are addressed through an “Info for GPs” page, which outlines the enrollment process for general practitioner clinics into GPFirst, ensuring that health care providers can participate and contribute to the initiative. Additionally, a dedicated web page helps visitors locate the nearest participating GPFirst clinic, facilitating easy access to services.

### GPFirst Awareness

To assess the campaign’s impact on community knowledge and awareness of GPFirst, we conducted 2 repeated cross-sectional surveys. The first cross-sectional survey (CS1) took place from July 2018 to August 2018, a year before the campaign started. The second survey (CS2) was conducted from November 2019 to December 2019, 4 months after the campaign began. Data collection was conducted by EA Research and Consulting Pte Ltd (EARC), a market research organization that was commissioned and compensated through the study grant. We measured awareness of GPFirst by asking participants in both CS1 and CS2 if they had heard of the program before participating in the surveys.

### GPFirst Knowledge

Knowledge about GPFirst was assessed through 3 specific questions (Multimedia Appendix 2) and was limited to those who reported being aware of GPFirst. Participants who answered all 3 questions correctly were categorized as “knowledgeable,” while those who did not were categorized as “not as knowledgeable.”

### Acceptability and Satisfaction of GPFirst

Acceptability was evaluated using responses from both CS1 and CS2, targeting 2 groups of participants: those who were aware of GPFirst and had visited a participating GPFirst clinic within the past 3 months (group A), and those who were unaware of GPFirst (group B). For group A, acceptability was assessed through three dichotomous yes or no questions: (1) whether they would revisit the participating GPFirst clinic, (2) whether they would recommend the clinic to others, and (3) whether their decision to visit the clinic was influenced by its participation in GPFirst (Multimedia Appendix 2). For group B, after receiving a brief overview of the program, acceptability was assessed by asking whether they would consider visiting participating GPFirst clinics before going to the ED in the future (Multimedia Appendix 1). Satisfaction with GPFirst was measured based on group A’s ratings of the overall experience with the program, using a 5-point Likert scale ranging from “very poor” to “excellent” (Multimedia Appendix 2).

### Recruitment

Quota sampling was used to recruit participants from three groups: (1) the ED group comprising of individuals who had visited CGH ED within the last 3 months, (2) the general practitioner group consisting of individuals who had visited general practitioners in eastern Singapore within the last 3 months, and (3) the public group composed of individuals who had not sought care at CGH ED or general practitioners in eastern Singapore within the last 3 months. If a participant had visited both general practitioner and CGH ED over the last 3 months, the most recent visit was used to determine the participant’s classification. Recruitment was conducted at public locations in eastern Singapore (Bedok, Pasir Ris, Tampines, and Simei), including train and bus stations, and food centers. A market research organization EARC was commissioned to help recruit participants and administer the surveys. EARC surveyors received training from the study team before being deployed to the designated areas between 11 AM and 9 PM, on both weekdays and weekends, to approach the public. To be eligible, participants were required to (1) be Singaporean citizens or permanent residents, (2) be aged 21 years and older, (3) be proficient in English, (4) not be employed by CGH, (5) have a residential address within eastern Singapore, and (6) belong to one of the three study groups.

### Sample Size Estimation

The sample size was determined using the Yamane formula with an error tolerance set at 0.05 [37], resulting in an estimated sample of 400 per study group, 1200 participants per survey, and a total of 2400 participants across both surveys.

### Statistical Analysis

Data analysis was performed using SPSS (version 27; IBM Corp), with a significance level of .05. Standard descriptive statistics including means, SDs, and frequencies were reported as appropriate. Chi-square, and where appropriate, Fisher exact tests were used to compare changes in outcomes between the CS1 and CS2 surveys. Analyses were also performed to examine the impact of the campaign across different age groups: (1) 21-39 years, (2) 40-59 years, and (3) 60 years and older [20], and multilogistic regression models adjusted for gender and ethnicity were performed to examine factors associated with GPFirst awareness. To address the potential inflation of type I error rates due to multiple comparisons, the false discovery rate (FDR) method was applied. The FDR method was chosen as it offers a more balanced approach, controlling false positives without being as overly conservative as the Bonferroni correction. While Bonferroni adjustment reduces the likelihood of type I errors, it also increases the risk of type II errors by being overly stringent [38]. The FDR method, on the other hand, maintains greater statistical power by allowing for a controlled proportion of false positives, making it more suitable for exploratory studies and public health research involving multiple hypothesis testing. Marital status was recoded into 2 groups: “married” and “single,” education level was recoded into 4 groups: “primary and below,” “secondary,” “preuniversity,” and “university and higher,” and residential type was recorded into 3 groups: “1-3 room public flats,” “4-5 room public flats,” and “private and others.” Participants who were part of the military service were excluded from the analysis. This exclusion was justified by studies suggesting that out-of-pocket costs can influence ED attendance [39]. Given that existing financing policies waive public hospital ED fees for military service personnel, their health-seeking behaviors may differ from those of other participants. This study was reported in accordance with the Strengthening the Reporting of Observational Studies in Epidemiology (STROBE) checklist (Multimedia Appendix 3).

### Ethical Considerations

This study was approved by the SingHealth Centralized Institutional Review Board (reference 2018/2196). Informed consent was obtained from all participants, and data were deidentified for analysis. Participants who completed the survey received an SGD $5 (US $3.73) supermarket voucher as a token of appreciation. Models present in images provided written informed consent to allow their image to be published.

## Results

### Overview

Within 5 months of the DMC rollout, the GPFirst Facebook page garnered 3162 likes, 3199 followers, and 38,404 engagements. An engagement is defined as a reaction, comment, share, or click on the GPFirst Facebook page [40]. The “#ThankYourGP” post achieved the highest number of views (n=24,602) and had the highest engagement (n=2618). Due to data limitations, the profiles of engaged individuals, or the platforms used (eg, smartphones, laptops) to access the Facebook page could not be analyzed in this study.

Data from 1191 participants for CS1 and 1161 participants for CS2 were analyzed (Table 1). Compared to CS1 participants, those in CS2 had higher proportions of women (54% vs 52%; *P*=.04), Chinese (65.5% vs 58.8%; *P*=.005), married individuals (66.3% vs 62%; *P*=.008), and those with higher education levels (*P*=.008). Additionally, CS2 participants had lower proportions of individuals with regular primary care physicians (*P*=.04). Participants’ characteristics stratified by age are detailed in Multimedia Appendix 4.

**Table 1:** Characteristics of participants from the precampaign (CS1^a^) and postcampaign (CS2^b^) surveys.

Variable		CS1 (n=1191)	CS2 (n=1161)
**Age groups (years), n (%)**			
	21-39	414 (34.8)	389 (33.5)
	40-59	467 (39.2)	471 (40.6)
	60 and older	310 (26)	301 (25.9)
**Gender, n (%)**			
	Women	572 (48)	653 (56.2)
	Men	619 (52)	508 (43.8)
**Ethnicity, n (%)**			
	Chinese	700 (58.8)	762 (65.6)
	Indian	78 (7)	74 (7)
	Malay	380 (31.9)	297 (25.6)
	Others	33 (3)	28 (2)
**Current marital status, n (%)**			
	Single	453 (38)	391 (33.7)
	Married	738 (62)	770 (66.3)
**Residential type, n (%)**			
	1-3 room public flats	316 (26.5)	283 (24.4)
	4-5 room public flats	730 (61.3)	724 (62.4)
	Private and others	145 (12.2)	154 (13.3)
**Highest education level, n (%)**			
	Primary and lower	239 (20.1)	173 (14.9)
	Secondary	445 (37.4)	440 (37.9)
	Preuniversity	297 (24.9)	319 (27.5)
	University and higher	210 (17.6)	229 (19.7)
**Regular primary care physician, n (%)**			
	Yes	674 (56.4)	608 (52.4)
	No	517 (43.6)	553 (47.6)

^a^CS1: cross-sectional survey 1 (baseline).

^b^CS2: cross-sectional survey 2.

### GPFirst Awareness

The proportion of participants who were aware of GPFirst in CS1 and CS2 was 11.5% (137/1191 participants) and 26.9% (312/1161 participants), respectively (Multimedia Appendix 5). This more than 2-fold increase in GPFirst awareness was also observed across all age groups. After adjustment (Table 2), the odds of being aware of GPFirst in CS2 were 2.7 times higher than in CS1 (odds ratio [OR] 2.69, 95% CI 2.15-3.37; *P*<.001). In each of the 3 age groups (ie, 21-39 years, 40-59 years, and 60 years and older), the odds of being aware GPFirst in CS2 were also 2.3 to 3.2 times higher than in CS1 (Table 3). Overall, married individuals had 1.3 times higher odds (95% CI 1.03-1.65, *P*=.03) of being aware of GPFirst compared to individuals who were single (Table 3). Higher education levels were also associated with GPFirst awareness, particularly among those aged 40-59 years. Additionally, individuals without a regular primary care physician had 1.8 times higher odds (95% CI 1.44-2.22; *P*<.001) of being aware of GPFirst compared to those with a regular primary care physician.

**Table 2:** Adjusted multilogistic regression analyses of factors associated with GPFirst awareness among participants from the precampaign (CS1^a^) and postcampaign (CS2^b^) surveys (N=2352).

Variable		Overall	
		Adjusted OR^c^ (95% CI)	*P* value
**Time**			
	CS1	1.00	—^d^
	CS2	2.64 (2.11-3.31)	<.001
**Marital status**			
	Single	1.00	—
	Married	1.31 (1.04-1.66)	.02
**Gender**			
	Men	1.00	—
	Women	1.16 (0.93-1.44)	.19
**Ethnicity**			0.27^e^
	Chinese	1.00	—
	Indian	0.89 (0.57-1.39)	.61
	Malay	0.94 (0.74-1.21)	.65
	Others	0.43 (0.18-1.01)	.05
**Education level**			< .001^e^
	Primary and lower	1.00	—
	Secondary	2.12 (1.47-3.05)	<.001
	Preuniversity	2.17 (1.47-3.20)	<.001
	University and higher	1.95 (1.28-2.97)	.002
**Residential type**			.21^e^
	1 to 3 room public flats	1.00	—
	4 to 5 room public flats	1.18 (0.90-1.56)	.23
	Private and others	1.41 (0.96-2.08)	.08
**Regular primary care physician**			
	Yes	1.00	—
	No	1.79 (1.44-2.22)	<.001

^a^CS1: cross-sectional survey 1 (baseline).

^b^CS2: cross-sectional survey 2.

^c^OR: odds ratio.

^d^Not applicable.

^e^Omnibus test.

**Table 3:** Age-stratified adjusted multilogistic regression analysis of factors associated with GPFirst awareness among participants from the precampaign (CS1^a^) and postcampaign (CS2^b^) surveys (N=2352).

Variable			21-39 age group					40-59 age group					60 and older age group		
			Adjusted OR^c^ (95% CI)		*P* value		Adjusted OR (95% CI)			*P* value		Adjusted OR (95% CI)			*P* value
**Time**															
	CS1	1.00		—^d^		1.00			—		1.00			—	
	CS2	3.17 (2.16-4.67)		<.001		2.33 (1.67-3.26)			<.001		2.92 (1.75-4.88)			<.001	
**Marital status**															
	Single	1.00		—		1.00			—		1.00			—	
	Married	1.33 (0.90-1.95)		.15		1.16 (0.76-1.80)			.49		1.73 (0.90-3.30)			.10	
**Gender**															
	Men	1.00				1.00			—		1.00			—	
	Women	1.20 (0.83-1.73)		.35		1.14 (0.82-1.59)			.44		1.24 (0.76-2.01)			.39	
**Ethnicity**					.36^e^					.21^e^					.34^e^
	Chinese	1.00		—		1.00			—		1.00			—	
	Indian	0.54 (0.25-1.18)		.12		1.34 (0.69-2.60)			.39		0.86 (0.31-2.41)			.77	
	Malay	0.84 (0.55-1.28)		.41		1.18 (0.80-1.72)			.51		0.53 (0.27-1.05)			.07	
	Others	0.50 (0.11-2.29)		.37		0.17 (0.02-1.26)			.08		0.94 (0.26-3.46)			.93	
**Education level**					.36^e^					.05^e^					.15^e^
	Primary and lower	1.00		—		1.00			—		1.00			—	
	Secondary	3.95 (0.49-31.95)		.20		2.18 (1.25-3.79)			.006		1.56 (0.88-2.76)			.13	
	Preuniversity	3.75 (0.46-30.45)		.22		1.97 (1.07-3.63)			.03		2.03 (0.97-4.24)			.06	
	University and higher	2.87 (0.35-23.58)		.33		1.96 (1.00-3.82)			.05		2.71 (0.94-7.86)			.07	
**Residential type**					.19^e^					.31^e^					.66^e^
	1 to 3 room public flats	1.00		—		1.00			—		1.00			—	
	4 to 5 room public flats	1.03 (0.64-1.65)		.91		1.36 (0.87-2.12)			.18		1.21 (0.68-2.16)			.51	
	Private and others	1.71 (0.87-3.35)		.12		1.55 (0.86-2.81)			.15		0.92 (0.39-2.16)			.85	
**Regular primary care physician**															
	Yes	1.00		—		1.00			—		1.00			—	
	No	1.85 (1.27-2.70)		.001		1.65 (1.19-2.28)			.003		2.05 (1.28-3.30)			.003	

^a^CS1: cross-sectional survey 1 (baseline).

^b^CS2: cross-sectional survey 2.

^c^OR: odds ratio.

^d^Not applicable.

^e^Omnibus test.

### GPFirst Knowledge

Among the 449 participants who self-reported being aware of GPFirst, significant differences were observed in the knowledge levels of GPFirst between CS2 and CS1 participants (Multimedia Appendix 6). The proportion of knowledgeable individuals was significantly higher in CS2 compared to CS1, particularly in the 21-39 age group (CS1: 19.6%; CS2: 66.7%; *P*<.001) and the 40-59 age group (CS1: 25.4%; CS2: 57.8%; *P*<.001). No significant difference in proportions was observed for the 60 years and older age group. A significantly higher proportion of CS2 participants correctly answered questions B (CS1: 38.0% vs CS2: 69.6%; *P*<.001) and C (CS1: 70.1% vs CS2: 87.2%; *P*<.001). No significant difference was observed for question A. After adjustment (Table 4), the odds of being knowledgeable about GPFirst were 4.2 times higher in CS2 than in CS1 (OR 4.20, 95% CI 2.62-6.73; *P*<.001). Compared to individuals with primary education or lower, those with secondary education and preuniversity education had 2.9 times (95% CI 1.35-6.17; *P*=.006) and 2.6 times (95% CI 1.14-5.70; *P*=.02) higher odds of being knowledgeable about GPFirst respectively. Additionally, individuals without a regular primary care physician had 1.5 times higher odds (95% CI 1.02-2.34; *P*=.04) of being knowledgeable about GPFirst compared to those with a regular primary care physician.

**Table 4:** Adjusted multilogistic regression analysis of factors associated with GPFirst knowledge among participants aware of GPFirst from the precampaign (CS1^a^) and postcampaign (CS2^b^) surveys (N=449).

Variable^c^		Adjusted OR^d^ (95% CI)	*P* value
**Time**			
	CS1	1.00	—^e^
	CS2	4.20 (2.62-6.73)	<.001
**Marital status**			
	Single	1.00	—
	Married	1.02 (0.65-1.59)	.94
**Gender**			
	Men	1.00	—
	Women	0.82 (0.55-1.24)	.35
**Ethnicity**			.14^f^
	Chinese	1.00	—
	Indian	0.50 (0.22-1.15)	.10
	Malay	0.82 (0.51-1.32)	.41
	Others	5.82 (0.57-59.73)	.14
**Education level**			.03^f^
	Primary and lower	1.00	—
	Secondary	2.88 (1.35-6.17)	.006
	Preuniversity	2.56 (1.14-5.70)	.02
	University and higher	1.90 (0.80-4.51)	.14
**Residential type**			.25^f^
	1 to 3 room public flats	1.00	—
	4 to 5 room public flats	0.76 (0.44-1.31)	0.32
	Private and others	1.21 (0.58-2.51)	0.61
**Regular primary care physician**			
	Yes	1.00	—
	No	1.54 (1.02-2.34)	0.04

^a^CS1: cross-sectional survey 1 (baseline).

^b^CS2: cross-sectional survey 2.

^c^Age was not included for adjustment due to multicollinearity with marital status.

^d^OR: odds ratio.

^e^Not applicable.

^f^Omnibus test.

### Acceptability and Satisfaction of GPFirst

Among the 166 participants who were aware of GPFirst and had visited participating GPFirst clinics in the past 3 months in both CS1 and CS2 (Multimedia Appendix 7), 60.2% (100/166 participants) chose these clinics specifically because they were part of GPFirst. Overall, 98.2% (163/166 participants) expressed their willingness to first visit participating GPFirst clinics in the future, and 95.2% (158/166 participants) said they would recommend participating GPFirst clinics to family and friends as a first stop before the ED. Regarding their overall experience with the GPFirst program, 87.3% (145/166 participants) rated it as “Good” or “Excellent.” Adjusted logistic regression analyses indicated that the 40-59 age group was more likely to choose participating GPFirst clinics because of the clinic’s involvement in the program compared to those in the younger age group (OR 2.33, 95% CI 1.13-4.82; *P*=.02; Multimedia Appendix 7).

Among the 1903 participants in both surveys who were unaware of GPFirst, 88.3% (1680/1903 participants) expressed their willingness to first visit a participating GPFirst clinic in the future. There was a significant association between age groups and the choice of visiting a participating GPFirst clinic (*P*<.001). Adjusted logistic regression (Multimedia Appendix 7) indicated that individuals aged 60 years and older were less likely to consider visiting a participating GPFirst clinic in the future compared to those aged 21-39 (OR 0.58, 95% CI 0.41-0.82) years (*P*=.002).

## Discussion

### Principal Findings

Our study found that awareness of GPFirst increased significantly from CS1 to CS2. Married individuals, those with higher education, and participants without a regular primary care physician were more likely to be aware of GPFirst. Knowledge also improved in CS2, particularly among younger age groups (21-39 years and 40-59 years). Among participants aware of GPFirst, 60.2% (100/166 participants) chose to visit their clinics because they were part of the program, 98.2% (163/166 participants) expressed willingness to return, and 95.2% (158/166 participants) would recommend these clinics. Satisfaction was high, with 87.3% (145/166 participants) rating their experience as “Good” or “Excellent.” However, those aged 40-59 years were less likely to choose clinics specifically due to GPFirst participation. Even among those unaware of GPFirst, 88.3% (1680/1903 participants) were willing to visit a participating clinic instead of the ED in the future. However, older participants (60 years and older) were less likely to consider GPFirst clinics compared to younger groups.

It is evident from our study that the DMC was an effective strategy in engaging the general community and increasing awareness and knowledge levels of GPFirst. Notably, the awareness of GPFirst among CS2 participants increased to 27%, which was 2.7 times higher than that of CS1 after adjusting for potential confounding differences between these 2 groups of participants. While 27% might seem modest compared to other Singapore national health campaigns such as the War on Diabetes, which achieved 61% exposure rates [41,42], it is in line with other awareness campaigns, like those for age-related macular degeneration, which achieved a 28% awareness rate after a 5-year campaign [43]. By leveraging digital platforms, particularly social media, which are widely accessed by diverse age cohorts in Singapore [22], the GPFirst campaign has succeeded in capturing the attention of a broad audience. Interestingly, even among participants in the 60 years and older age group, which typically exhibits lower DM use [22], the odds of being GPFirst aware in the CS2 group was 2.92 compared to the CS1 group. Our findings also revealed an interesting association between not having a regular primary care physician and a higher likelihood of being aware of GPFirst across diverse age groups. The reason for this was unclear in this study and warrants further investigation in future studies. Within our study cohort, a substantial proportion (47.2%) do not have a regular primary care physician. This suggests the potential need for future GPFirst publicity campaigns to include educational elements emphasizing the importance of having a regular primary care physician. Encouraging individuals to have a regular primary care physician can lead to well-documented benefits, such as decreased health care use [44,45]. Additionally, this move will align GPFirst with Singapore’s Healthier SG initiative, a national effort by the Ministry of Health to promote preventive care and empower patients to take steps toward better health [46]. A key aspect of this initiative is to encourage all residents to enroll with a regular family doctor who serves as the first point of contact for health management.

Our results also offered evidence supporting the effectiveness of the campaign in enhancing knowledge levels about GPFirst among the target population. This suggests that DM-driven health promotion campaigns can deliver reliable and trustworthy information to the general public helping to combat misinformation [47-50]. Despite the improvement in knowledge from CS1 to CS2 regarding general practitioners participating in the GPFirst program, 30% (41/137 respondents) still answered the criteria about subsidy eligibility incorrectly. This highlights the need for targeted content to address misconceptions about this aspect in future campaign designs.

The increased proportion of GPFirst knowledgeable respondents among the 21-39 years and 40-59 years age groups suggests that the campaign delivered relevant health promotion and educational materials that resonated with this demographic, potentially leading to a better understanding of GPFirst among young to middle-aged adults. This success might be partially due to our content design and production, which were guided by evidence-based models such as the Andersen model. The 60 years and older age group did not show a statistically significant increase in GPFirst knowledgeable respondents between CS1 and CS2. It is possible that the campaign content was more aligned with the preferences and digital behavior of younger participants, resulting in a smaller impact on older adults. However, the 12% increase in knowledgeable respondents in this age group still reflects some success in engagement, which is important given that this age group contributed to about 15% of nonurgent ED visits [20]. These findings underscore the importance of tailoring health campaigns to effectively target different age groups, ensuring that accurate health information is accessible and well-received by diverse populations [51]. This approach is crucial for the success of public health initiatives in reaching and educating all segments of the community. Similar to our findings on GPFirst awareness, our results also revealed an unexpected association between not having a regular primary care physician and a higher likelihood of being knowledgeable about GPFirst. This should also be addressed in future investigations.

Upon being introduced to the GPFirst program during the survey to participants who were previously unaware of GPFirst, 88% of them expressed their willingness to consider first visiting a participating GPFirst clinic instead of the ED in the future. Significant associations were observed for age groups and choice of health care providers. Older adults who were unaware of GPFirst still have an inclination preference toward EDs even after being introduced to GPFirst. This preference could be due to barriers in accessing primary care, which could include challenges related to transportation and mobility [52], and an expectation for more timely and specialized care [53,54]. Clearly, this suggests the need to have age-dependent strategies to further improve the uptake of participating GPFirst clinics, particularly among older residents.

Among individuals who visited a participating GPFirst clinic within the last 3 months during the study periods, high levels of acceptability of GPFirst were observed across all age groups. Nearly all respondents expressed their willingness to visit the participating GPFirst clinic again. About 95% also indicated their intention to recommend their family or friends to seek care at the participating GPFirst clinic before the ED in the future. The high percentage of participants who rated their GPFirst experience as “good” or “excellent” also suggests a high satisfaction level of their respective GPFirst experience. Among survey participants who were aware of GPFirst and who visited participating GPFirst clinics in the past 3 months of the study periods, only approximately 60% of participants indicated that they chose to visit their general practitioner clinics because they were participating in GPFirst clinics, with those aged 40-59 years more likely to cite GPFirst participation as a reason for their clinic choice. Evidently, these results highlighted 2 important points. First, the fact that about 40% of individuals who were GPFirst aware did not visit the participating GPFirst clinics because of their affiliation to GPFirst suggests that benefits (eg, financial subsidy and higher priority at ED) offered by GPFirst may not be their key considerations when they decided to visit these participating clinics. Insights from previous qualitative studies may provide possible explanations for this finding [55,56]. While financial subsidies are an important factor, other factors such as positive and trusting general practitioner–patient relationships, clinical accessibility, and interpersonal influences, such as advice from family and friends were also observed to play important roles in shaping health-seeking decisions. Second, the findings also highlighted again the need to have age-dependent strategies to further improve the uptake of participating GPFirst clinics among residents while accounting for other relevant determinants that may drive health-seeking behavior among residents with nonurgent medical conditions [56].

### Public Health Implications

The success of the “#ThankYourGP” campaign, which achieved the highest engagement among all post categories, demonstrated the public health potential of user-generated content in fostering interaction on social media, and as a tool for health communication and advocacy. Digital platforms offer a participatory model of engagement, where individuals share personal experiences and narratives, fostering engagement, community-driven health promotion, and peer-to-peer knowledge exchanges [13,57,58]. Beyond increasing engagement and content dissemination, DMCs have demonstrated their ability to drive behavioral change [13,57]. Given the growing reliance on DM for health information, policy makers and health agencies can leverage DMCs as part of broader health promotion strategies, extending their use beyond awareness-building initiatives to influence longer-term health behaviors and service use.

The effectiveness of a DM-driven campaign in raising awareness and knowledge about GPFirst highlights the growing role of digital platforms as essential tools for public health education. Social media and digital platforms allow rapid, scalable, and cost-effective dissemination of information [58]. These platforms can improve accessibility to health information, especially in underserved communities and health systems where traditional media may have limited reach and younger populations who primarily consume health-related content digitally [15,16,24,58]. As digital engagement continues to shape public health communication, integrating such content into national health initiatives could improve health literacy, service use, and preventive health behaviors. Furthermore, algorithm-driven content distribution enables the delivery of tailored health messages to specific demographic groups based on their digital behavior, interests, and search patterns. Health policy makers should consider expanding DMCs beyond awareness and knowledge-building to facilitate behavior change. For instance, linking social media content with telehealth services, appointment scheduling, or digital screening tools to enable seamless access with a single click [58].

The findings of acceptability and satisfaction are particularly relevant to Singapore, given its distinctive health care financing structure and emphasis on public-private partnerships. Singapore’s financing system, characterized by a combination of government subsidies, mandatory savings schemes like MediSave, and private insurance, creates a health care landscape that differs from many other systems [59]. These structural elements shape how individuals perceive and engage with health care programs, including initiatives like GPFirst. Additionally, Singapore’s emphasis on public-private partnerships facilitates collaboration between government health care providers and private providers like general practitioners, this may serve as a model for other similar health care systems, where alignment and partnerships between public and private providers could improve appropriate health-seeking behaviors [60]. However, scalability beyond Singapore requires consideration of local health system structures [46,55,56]. For health care systems with strong hospital-community partnerships, DMCs for similar programs like GPFirst may be more effective when integrated into primary care outreach programs or coordinated with community public health workers to maximize impact. In settings with fragmented health care systems or high out-of-pocket costs, awareness-building DMCs alone may have limited effectiveness due to existing barriers to care access. In such contexts, additional policy interventions may be necessary to encourage changes in health-seeking behaviors.

### Limitations

There are several limitations in our study that should be acknowledged. First, the COVID-19 pandemic significantly impacted ED visits, making it difficult to reliably obtain rates of nonurgent ED visits after the rolling out of the DM-driven GPFirst campaign. The confirmation of the first COVID-19 case in Singapore on January 23, 2020, followed by the development of multiple local transmission clusters and the implementation of a lockdown in April 2020, prevented us from conducting a meaningful comparison of nonurgent ED visit rates before and after the roll-out of DM-driven GPFirst campaign. Second, the sample of individuals with a recent encounter with a participating GPFirst clinic was relatively small. Therefore, their perceptions of acceptability and satisfaction with GPFirst may not be fully representative of the past GPFirst patients’ views and experiences. Third, the inclusion of only English-literate participants may affect the generalizability of results to Singapore's multicultural society. Finally, it is important to note that this study used a convenience sample at 2 different time points, with recruitment conducted at public locations in eastern Singapore. This approach may have introduced selection bias, as individuals who frequent public places might differ systematically from those who do not, such as housebound individuals or those who rely on private transportation. Additionally, variations in participant characteristics across the 2 time points, as well as potential self-selection bias (where those more interested or available chose to participate), could have influenced the findings. To address this, a more rigorous longitudinal study design using household surveys would be preferable to track changes in knowledge, awareness, and behavior over time.

### Conclusions

This study has offered evidence that a carefully crafted DM-driven campaign can have a positive impact on the awareness and knowledge of GPFirst among the target population across all age groups. Our surveys show high population-wide acceptance of GPFirst and high satisfaction with GPFirst among the program participants. The findings also highlighted the need to have age-dependent strategies to further improve participation among residents. Overall, our experience does suggest that a publicity campaign that uses a mix of social media platforms (Facebook) and digital methods may be adopted for other public health campaigns with similar objectives of modifying the population’s health-seeking behaviors through public health education and promotion efforts.

## Appendix

Multimedia Appendix 1 GPFirst Facebook Posts.

Multimedia Appendix 2 GPFirst survey.

Multimedia Appendix 3 STROBE (Strengthening the Reporting of Observational Studies in Epidemiology) checklist.

Multimedia Appendix 4 Survey participants’ characteristics stratified by age groups.

Multimedia Appendix 5 Comparison of GPFirst awareness levels.

Multimedia Appendix 6 Comparison of correct response rates for each knowledge question.

Multimedia Appendix 7 Acceptability and satisfaction of GPFirst.

## Abbreviations

CGH: Changi General Hospital
CS1: cross-sectional survey 1
CS2: cross-sectional survey 2
DM: digital media
DMC: digital media campaign
EARC: EA Research and Consulting Pte Ltd
ED: emergency department
FDR: false discovery rate
OR: odds ratio
STROBE: Strengthening the Reporting of Observational Studies in Epidemiology
