# Supplementary material for: Evaluation of a Digital Media Campaign to Promote Knowledge and Awareness of the GPFirst Program for Nonurgent Conditions: Repeated Survey Study
Source: JMIR Public Health Surveill. 2025 Apr 14;11:e66062. doi: 10.2196/66062 (PMC12038294; doi:10.2196/66062)

# Multimedia Appendix 1 – GPFirst Facebook Posts

Digital media publicity materials.


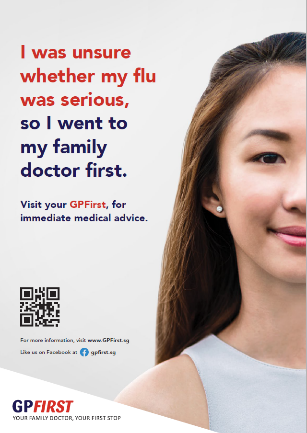

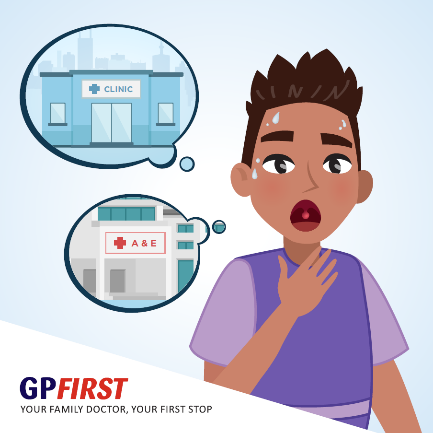


Facebook post for #ThankYourGP.


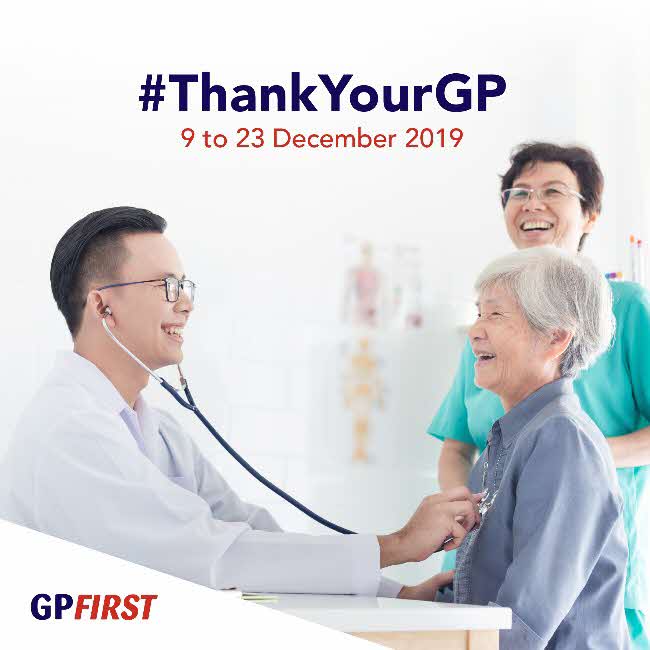

Supplement: Multimedia Appendix 1 [file publichealth_v11i1e66062_app1.docx]
