# Supplementary material for: Evaluation of a Digital Media Campaign to Promote Knowledge and Awareness of the GPFirst Program for Nonurgent Conditions: Repeated Survey Study
Source: JMIR Public Health Surveill. 2025 Apr 14;11:e66062. doi: 10.2196/66062 (PMC12038294; doi:10.2196/66062)
Supplement: Multimedia Appendix 2 [file publichealth_v11i1e66062_app2.docx]

# Multimedia Appendix 2 – GPFirst survey.

**Visit History** (choose one only; if both 1 and 2 are valid, choose the one that corresponded to the latest visit):

1. Visited CGH A&E over the last 3 months
2. Visited GP clinics in eastern part of Singapore over the last 3 months
3. None of the above

**Regular Primary Care Physician**

Do you have a regular family doctor/GP whom you will consult when you have a health problem?

1. Yes 2. No

**Sex**: 1. Male 2. Female

**Race**: 1. Chinese 2. Malay 3. Indian 4. Others (please specify :______________)

**Age** :

1. 21 -29 years 4. 50-59 years
2. 30- 39 years 5. 60-64 years
3. 40-49 years 6. 65 years and above

**Marital status**:

1. Single 2. Married 3. Divorced/Separated 4. Widow/Widower

**Highest education level**:

1. No formal education 4. A Level / Diploma
2. Primary / PSLE 5. University degree and above
3. N-Level / O-Level / ITE

**Occupation**: ___________________________________________

1. Top Management (CEO, MD, GM, VP, Director, etc.)
2. Middle Management (department manager, supervisory level)
3. Executive
4. Clerical (Clerks, Admin Assistant etc.)
5. Blue-collar (Construction, Renovation, Hawker, Driver, Technician etc.)
6. Professionals (Doctor, Lawyer, Architect, Consultant, Engineer etc.)
7. Free-lancer (Tuition / Music Teacher, Artist, Designer, etc.)
8. Housewife
9. Student
10. Retired
11. Sole Proprietor / Businessman
12. Others (please specify ________________________________)

**Housing type**:

1. HDB 1-2 rooms 4. HDB 5 or more rooms 7. Landed Property

2. HDB 3 rooms 5. Mansionette / HUDC 8. Others (pls specify: ____________)

3. HDB 4 rooms 6. Pte Apt / Condominium

# Three knowledge questions

1. Have you heard of the GPFirst Programme before participating in this survey?
   1. Yes <go to question 2>
   2. No <go to Section 2>
2. Complete the following table, indicating whether the statements about GPFirst programme are True or False

| **No** | **Statement** | **True** | **False** |
| --- | --- | --- | --- |
| a. | GPFirst programme aims to encourage patients with mild and moderate conditions (e.g. flu, fever, small cuts, stomach, etc.) to seek treatment by a general practitioner (GP) instead of directly seeking treatment at the Accident and Emergency (A&E) department at a public hospital. |  |  |
| b. | Patients who are referred to A&E by any private GP clinic will get a $50 subsidy on their respective A&E attendance fees when they subsequently visit the A&E. |  |  |

1. The GPFirst programme is currently applicable at the A&Es of the following public hospitals (Please tick all that is applicable)

Alexandra Hospital

Changi General Hospital

National University Hospital

Ng Teng Fong General Hospital

Singapore General Hospital

Tan Tock Seng Hospital

# Acceptability

Group A: those who were aware of GPFirst and had visited a participating GPFirst clinic within the past three months.

1. The following statements seek to understand your satisfaction level of GPFirst programme based on your last experience at a GPFirst clinic using a scale from 5 to 1, where 5 = Excellent, 4 = Good, 3 = Satisfactory, 2 = Poor, 1 = Very Poor. <<If Answer=1, or 2, please state reason so that the we can take action to improve the programme>>

| **Statement** | **Satisfaction Level** | | | | |
| --- | --- | --- | --- | --- | --- |
|  | **Excellent** | **Good** | **Satisfactory** | **Poor** | **Very Poor** |
| 1. Overall experience of GPFirst programme   Reason:______________________________________________________ | 5 | 4 | 3 | 2 | 1 |

1. Would you consider visiting the same GPFirst participating clinic again prior to any visit to A&E in future?
2. Yes
3. No
4. Would you recommend your family/friends to visit the same GPFirst participating clinic prior to any visit to A&E in future?
5. Yes
6. No
7. Have you chosen to visit the GPFirst clinic over other clinics because the clinic is participating in the GPFirst programme?
8. Yes
9. No

Group B: Participants who were unaware of GPFirst

**Overview of GPFirst Programme**

- This programme aims to encourage patients with mild and moderate conditions (e.g. flu, fever, small cuts, stomach, etc.) to seek treatment by a general practitioner (GP) instead of directly seeking treatment at the Accident and Emergency (A&E) department at a public hospital.
- Patients who are referred to A&E by GPFirst participating clinic will get a $50 subsidy on their respective A&E attendance fees when they subsequently visit the A&E.

<< Participants may refer to the list of existing participating GPFirst clinics if necessary>>

1. Would you consider visiting a GPFirst participating clinic prior to any visit to CGH A&E in future?
2. Yes
3. No
