# Supplementary material for: Evaluation of a Digital Media Campaign to Promote Knowledge and Awareness of the GPFirst Program for Nonurgent Conditions: Repeated Survey Study
Source: JMIR Public Health Surveill. 2025 Apr 14;11:e66062. doi: 10.2196/66062 (PMC12038294; doi:10.2196/66062)
Supplement: Multimedia Appendix 4 [file publichealth_v11i1e66062_app4.docx]

# Multimedia Appendix 4 – Survey participants’ characteristics stratified by age groups.

| Variable | 21 – 39  (n = 803) | 40 – 59  (n = 938) | 60 and above  (n = 611) |
| --- | --- | --- | --- |
| **Sex, n (%)** |  |  |  |
| Female | 400 (49.8) | 535 (57.0) | 337 (55.2) |
| Male | 403 (50.2) | 403 (43.0) | 274 (44.8) |
| **Ethnicity, n (%)** |  |  |  |
| Chinese | 456 (56.8) | 588 (62.7) | 418 (68.4) |
| Malay | 267 (33.3) | 272 (29.0) | 138 (22.6) |
| Indian | 62 (7.7) | 55 (5.9) | 35 (5.7) |
| Others | 18 (2.2) | 23 (2.5) | 20 (3.3) |
| **Current Marital Status, n (%)** |  |  |  |
| Single | 508 (63.3) | 201 (21.4) | 135 (22.1) |
| Married | 295 (36.7) | 737 (78.6) | 476 (77.9) |
| **Residential Type, n (%)** |  |  |  |
| 1-3 room public flats | 174 (21.7) | 239 (25.5) | 186 (30.4) |
| 4-5 room public flats | 535 (66.6) | 564 (60.1) | 355 (58.1) |
| Private and others | 94 (11.7) | 135 (14.4) | 70 (11.5) |
| **Highest education level, n (%)** |  |  |  |
| Primary and below | 15 (1.9) | 158 (16.8) | 239 (39.1) |
| Secondary | 226 (28.1) | 397 (42.3) | 262 (42.9) |
| Pre-university | 310 (38.6) | 227 (24.2) | 79 (12.9) |
| University and above | 252 (31.4) | 156 (16.6) | 31 (5.1) |
| **Regular Primary Care Physician, n (%)** |  |  |  |
| Yes | 402 (50.1) | 536 (57.1) | 344 (56.3) |
| No | 401 (49.9) | 402 (42.9) | 267 (43.7) |
