# Supplementary material for: Evaluation of a Digital Media Campaign to Promote Knowledge and Awareness of the GPFirst Program for Nonurgent Conditions: Repeated Survey Study
Source: JMIR Public Health Surveill. 2025 Apr 14;11:e66062. doi: 10.2196/66062 (PMC12038294; doi:10.2196/66062)
Supplement: Multimedia Appendix 5 [file publichealth_v11i1e66062_app5.docx]

# Multimedia Appendix 5 – Comparison of GPFirst awareness levels among participants in the pre-campaign (CS1) and post-campaign (CS2) groups, overall and stratified by age.

|  | | | CS1^a^ | CS2 | *P*-value | FDR *P*-value |
| --- | --- | --- | --- | --- | --- | --- |
| **Aware of GPFirst** | | | | | | |
| **Overall, n (%)** | | | 1,191 | 1,161 |  |  |
|  | Yes | | 137 (11.5) | 312 (26.9) | < .001 | < .001 |
|  | No | | 1,054 (88.5) | 849 (73.1) |  |  |
| **21 – 39 years old, n (%)** | | | 414 | 389 |  |  |
|  | Yes | | 46 (11) | 109 (28) | < .001 | < .001 |
|  | No | | 368 (88.9) | 280 (72) |  |  |
| **40 – 59 years old, n (%)** | | | 467 | 471 |  |  |
|  | | Yes | 67 (14) | 135 (28.6) | < .001 | < .001 |
|  | | No | 400 (85.7) | 336 (71.3) |  |  |
| **60 years old and older, n (%)** | | | 310 | 301 |  |  |
|  | | Yes | 24 (8) | 68 (23) | < .001 | < .001 |
|  | | No | 286 (92.3) | 233 (77.4) |  |  |

**^a^CS1**: baseline cross-sectional survey, **CS2**: second cross-sectional survey
