# Supplementary material for: Evaluation of a Digital Media Campaign to Promote Knowledge and Awareness of the GPFirst Program for Nonurgent Conditions: Repeated Survey Study
Source: JMIR Public Health Surveill. 2025 Apr 14;11:e66062. doi: 10.2196/66062 (PMC12038294; doi:10.2196/66062)
Supplement: Multimedia Appendix 6 [file publichealth_v11i1e66062_app6.docx]

# Multimedia Appendix 6 - Comparison of correct response rates for each knowledge question among participants who aware of GPFirst in the pre-campaign (CS1) and post-campaign (CS2) groups, overall and stratified by age.

|  | | CS1^a^ | CS2 | *P*-value | FDR *P*-value |
| --- | --- | --- | --- | --- | --- |
| **Knowledge levels of GPFirst** | | | | | |
| **Overall, n (%)** | | 137 | 312 |  |  |
|  | All questions correctly answered (‘knowledgeable’) | 36 (26.3) | 186 (59.8) | < .001 | < .001 |
|  | ≥ 1 question incorrectly answered (‘not as knowledgeable’) | 101 (73.7) | 125 (40.2) |  |  |
| **Question A^b^, n (%)** | |  |  | .045 | .05 |
|  | Correct | 137 (100.0) | 303 (97.1) |  |  |
|  | Incorrect | 0 | 9 (3) |  |  |
| **Question B^c^, n (%)** | |  |  | < .001 | < .001 |
|  | Correct | 52 (38) | 217 (69.6) |  |  |
|  | Incorrect | 85 (62) | 95 (30) |  |  |
| **Question C^d^, n (%)** | |  |  | <.001 | < .001 |
|  | Correct | 96 (70) | 272 (87.2) |  |  |
|  | Incorrect | 41 (30) | 40 (13) |  |  |
| **21 – 39 years old, n (%)** | | 46 | 109 |  |  |
|  | Knowledgeable | 9 (20) | 72 (66) | < .001 | < .001 |
|  | Not as knowledgeable | 37 (80) | 37 (34) |  |  |
| **40 – 59 years old, n (%)** | | 67 | 135 |  |  |
|  | Knowledgeable | 17 (25) | 78 (58) | < .001 | < .001 |
|  | Not as knowledgeable | 50 (75) | 57 (42) |  |  |
| **60 years old and older, n (%)** | | 24 | 68 |  |  |
|  | Knowledgeable | 10 (42) | 36 (53) | .31 | .31 |
|  | Not as knowledgeable | 14 (58) | 32 (47) |  |  |

**^a^CS1**: baseline cross-sectional survey, **CS2**: second cross-sectional survey

**^b^Question A**: GPFirst programme aims to encourage patients with mild and moderate conditions (e.g. flu, fever, small cuts, stomach, etc.) to seek treatment by a general practitioner (GP) instead of directly seeking treatment at the emergency department (ED) at a public hospital. (True/False)

**^c^Question B**: Patients who are referred to ED by any private GP clinic will get a $50 subsidy on their respective ED attendance fees when they subsequently visit the ED. (True/False)

**^d^Question C**: The GPFirst programme is currently applicable at the ED of the following public hospitals: (Select all applicable options: Alexandra Hospital; Changi General Hospital; National University Hospital; Ng Teng Fong General Hospital; Singapore General Hospital; Tan Tock Seng Hospital)
