# Supplementary material for: Evaluation of a Digital Media Campaign to Promote Knowledge and Awareness of the GPFirst Program for Nonurgent Conditions: Repeated Survey Study
Source: JMIR Public Health Surveill. 2025 Apr 14;11:e66062. doi: 10.2196/66062 (PMC12038294; doi:10.2196/66062)
Supplement: Multimedia Appendix 7 [file publichealth_v11i1e66062_app7.docx]

# Multimedia Appendix 7 - Acceptability and satisfaction with GPFirst among participants from the pre-campaign (CS1) and post-campaign (CS2) surveys, presented overall and stratified by age.

| **Participants who were aware of GPFirst and visited GPFirst clinics in the past 3 months** | | Overall  N = 166 | 21 – 39  N = 59 | 40 – 59  N = 70 | 60 and older  N = 37 | *P*-value | FDR *P*-value |
| --- | --- | --- | --- | --- | --- | --- | --- |
| **Would consider visiting GPFirst clinic prior to ED in the future^a^, n, (%)** | |  |  |  |  |  |  |
|  | Yes | 163 (98.2) | 56 (94.9) | 70 (100.0) | 37 (100.0) | .05 | .08 |
|  | No | 3 (1.8) | 3 (5.1) | 0 | 0 |  |  |
| **Would recommend family/friends to visit GPFirst clinic prior to ED in the future^a^, n, (%)** | |  |  |  |  |  |  |
|  | Yes | 158 (95.2) | 55 (93.2) | 68 (97.1) | 35 (94.6) | .58 | .58 |
|  | No | 8 (4.8) | 4 (6.8) | 2 (2.9) | 2 (5.4) |  |  |
| **Chose to visit the clinic because it is participating in GPFirst^a^, n, (%)** | |  |  |  |  |  |  |
|  | Yes | 100 (60.2) | 28 (47.5) | 48 (68.6) | 24 (64.9) | .04 | .10 |
|  | No | 66 (39.8) | 31 (52.5) | 22 (31.4) | 13 (35.1) |  |  |
| **Experience of GPFirst^a^, n, (%)** | |  |  |  |  |  |  |
|  | Satisfactory | 21 (12.7) | 4 (6.8) | 12 (17.1) | 6 (16.2) | .15 | .19 |
|  | Good | 100 (60.2) | 34 (57.6) | 40 (57.1) | 26 (70.3) |  |  |
|  | Excellent | 45 (27.1) | 21 (35.6) | 18 (25.7) | 5 (13.5) |  |  |
| **Participants who were unaware of GPFirst** | | Overall  N = 1903 | 21 – 39  N = 648 | 40 – 59  N = 736 | 60 and older  N = 519 |  |  |
| **Would consider visiting a GPFirst clinic prior to ED in the future^b^, n, (%)** | |  |  |  |  |  |  |
|  | Yes | 1680 (88.3) | 584 (90.1) | 662 (89.9) | 434 (83.6) | < .001 | < .001 |
|  | No | 223 (11.7) | 64 (9.9) | 74 (10.1) | 85 (16.4) |  |  |

^a^These questions were directed to individuals who were aware of GPFirst and had a GPFirst clinic visit within the last three months.

^b^This question was directed to all individuals in both surveys who were unaware of GPFirst

Adjusted multi logistic regression analysis of factors associated with visiting a GPFirst clinic due to its involvement in GPFirst, among participants from the CS1 and CS2 surveys (N = 166).

| Variable | | Adjusted OR (95% CI) | P-value |
| --- | --- | --- | --- |
| **Age** | |  | .047^a^ |
|  | 21 – 39 | 1.00 |  |
|  | 40 – 59 | 2.33 (1.13 to 4.82) | .02 |
|  | 60 and above | 2.23 (0.93 to 5.38) | .07 |
| **Gender** | |  |  |
|  | Male | 1.00 |  |
|  | Female | 0.99 (0.51 to 1.92) | .98 |
| **Ethnicity** | |  | .77^a^ |
|  | Chinese | 1.00 |  |
|  | Malay | 0.86 (0.41 to 1.79) | .68 |
|  | Indian | 2.15 (0.41 to 11.40) | .37 |
| **Residential Type** | |  | .79^a^ |
|  | 1 – 3 room | 1.00 |  |
|  | 4 – 5 room | 0.80 (0.30 to 2.17) | .67 |
|  | Private or others | 1.05 (0.31 to 3.53) | .94 |

^a^Omnibus test

Adjusted multi logistic regression analysis of factors associated with the intention to visit a GPFirst clinic in the future among those unaware of GPFirst from the CS1 and CS2 surveys (N = 1903)

| Variable | | Adjusted OR (95% CI) | P-value |
| --- | --- | --- | --- |
| **Age** | |  | .001^a^ |
|  | 21 – 39 | 1.00 |  |
|  | 40 – 59 | 0.99 (0.70 to 1.42) | .97 |
|  | 60 and above | 0.58 (0.41 to 0.82) | .002 |
| **Gender** | |  |  |
|  | Male | 1.00 |  |
|  | Female | 1.52 (1.15 to 2.02) | .004 |
| **Ethnicity** | |  | .29^a^ |
|  | Chinese | 1.00 |  |
|  | Malay | 1.13 (0.82 to 1.57) | .45 |
|  | Indian | 1.80 (0.89 to 3.63) | .10 |
|  | Others | 0.76 (0.36 to 1.59) | .46 |
| **Residential Type** | |  | .09^a^ |
|  | 1 – 3 room | 1.00 |  |
|  | 4 – 5 room | 1.41 (1.03 to 1.94) | .03 |
|  | Private or others | 1.32 (0.81 to 2.14) | .27 |

^a^Omnibus test
